# It’s not raining men: a mixed-methods study investigating methods of improving male recruitment to health behaviour research

**DOI:** 10.1186/s12889-019-7087-4

**Published:** 2019-06-24

**Authors:** Jillian Ryan, Luke Lopian, Brian Le, Sarah Edney, Gisela Van Kessel, Ronald Plotnikoff, Corneel Vandelanotte, Tim Olds, Carol Maher

**Affiliations:** 1 Commonwealth Scientific and Industrial Research Organisation, Adelaide, Australia; 2 Alliance for Research in Exercise, Nutrition and Activity: University of South Australia, Adelaide, Australia; 3 Priority Research Centre for Physical Activity and Nutrition, The University of Newcastle, Newcastle, Australia; 4 Physical Activity Research Group, School of Human Health and Social Sciences, Central Queensland University, Rockhampton, Australia

**Keywords:** Online social networks, Male, Men’s health, Physical activity, Randomised controlled trial, Health behaviour, Recruitment, Facebook

## Abstract

**Background:**

Although gender is an important determinant of health behaviour with males less likely to perform health-protective behaviours, samples in health behaviour research are heavily biased towards females. This study investigated the use of online social network, Facebook, to reach and recruit inactive males to a team-based, social, and gamified physical activity randomised controlled trial.

**Methods:**

Methodological techniques included a narrative literature review, survey of inactive males (*n* = 34) who rated advertisement images and text captions on scales of 1–10, and trial Facebook-delivered recruitment campaigns. Advertisement effectiveness was measured by cost-per-click to the study website, number of expressions of interest, and study enrolments from males.

**Results:**

Survey results showed that vibrant images of men exercising accompanied by concise captions (< 35 words) were most effective. An advertising campaign incorporating these components achieved a cost-per-click of $0.60, with 80% of *n* = 50 expressions of interest being from men, a marked improvement from baseline campaigns in which only 11% of expressions of interest were from men. Despite this, men who were recruited through the targeted campaign failed to enrol into the study, primarily due to reluctance to invite friends to join their team. An alternative strategy of encouraging females to invite men boosted male participation from 18% of the sample at baseline to 29% in the targeted recruitment phase.

**Conclusions:**

Evidence-based approaches can improve Facebook recruitment outcomes, however, there are complex barriers hindering male recruitment to health behaviour studies that may necessitate multi-faceted strategies including involvement of family and friends.

**Electronic supplementary material:**

The online version of this article (10.1186/s12889-019-7087-4) contains supplementary material, which is available to authorized users.

## Background

Physical inactivity is a leading modifiable risk factor for chronic disease [1], yet approximately two-thirds of adults fail to meet recommended physical activity targets [2]. Gender is an important determinant of health-risk and health-promoting behaviours [3], with males being more likely to perform high-risk behaviours including smoking, unhealthy eating, excess alcohol consumption, and physical inactivity [4], and less likely to seek medical and psychological help [5] or participate in health-promotion programs [6]. Reviews further suggest that males comprise only about 20% of health behaviour research samples [7], contributing to a lack of evidence on how to increase men’s uptake of health-promoting behaviours [8].

The internet and online social networks (OSNs) are gaining interest as platforms for promoting engagement with health behaviour initiatives and research [9]. OSNs can provide an interactive and engaging medium to deliver health promotion content, which is positively associated with longer lasting intervention effects [10]. Research shows that use of OSNs is positively associated with sedentary behaviour [11], suggesting that such platforms may facilitate access to inactive populations, who are in greatest need of health behaviour interventions. Facebook is the largest of the OSNs, with 2.07 billion active users worldwide [12], 56% of which are men [13]. It therefore offers broad reach as a recruitment platform, whilst simultaneously allowing content to be targeted to specific users based on their demographic characteristics and geographical location.

Employing OSNs as recruitment platforms for health behaviour research is growing in popularity [14, 15]. Preliminary evidence generally supports OSNs as effective and cost-efficient platforms for targeting specific population subgroups [11, 16], but that the effectiveness of such campaigns is affected by advertisement characteristics such as message framing and image choice [17]. For example, Choi et al. [17] found that males’ level of engagement with Facebook advertising materials aiming to recruit participants to an online mental health study was greater when advertisements emphasised a ‘strength’ message (e.g. “how tough is your mind? Find out your psychological strength…”), when compared to a 'resilience' (e.g. “How resilient are you?”) or 'happiness' (e.g. “How happy are you?”) message. To maximise the potential of OSNs as research recruitment and health promotion platforms, there is a need for further evidence regarding the design of effective campaign content, particularly to reach males. The current study aims to contribute to this emerging body of research by addressing the following research questions:

1. What evidence exists to guide the development of online health advertisements for men?
2. How do men respond to image and text-based components of health advertisements?
3. Can an evidence-based advertising campaign boost interest, and enrolment, into an OSN-based physical activity program?

## Methods

This study took place within and alongside a large randomised controlled trial (RCT) evaluating a social and gamified physical activity intervention delivered via mobile phone application (app) called Active Team. Active Team users participate in clusters with between three and eight of their existing Facebook friends and use a pedometer to measure their daily step counts, which they then record in the app. Further detail regarding the Active Team RCT research methodology can be found elsewhere [18]. Ethical approval for the RCT and the current study (protocol number: P060–10) was granted by the University of South Australia’s Human Research Ethics Committee. Informed consent was obtained from all participants on page 1 of the online survey where participants indicated whether they had understood the participant information sheet and agreed to participate.

Active Team is currently undergoing evaluation in a RCT, which aimed to recruit 440 inactive adults with an approximately equal balance of male and females. Eligibility criteria required that all participants were aged between 18 and 65 years old, self-reported to be insufficiently active (< 150 min of moderate-vigorous physical activity per week), regular users of Facebook, and able to assemble or join a team with at least two other participants. To achieve the necessary cluster structure, ‘seed’ participants were recruited via Facebook in the first instance and encouraged to recruit at least two of their Facebook friends to form an eligible team. Initial/baseline recruitment campaigns resulted in more than 1600 formal expressions of interest (EOIs), however, the vast majority (> 80%) of these were from females, leading to a marked gender imbalance in our sample partway through recruitment.

### Outcomes

This study has three outcomes of interest:

1. Cost per click (CPC), referring to the monetary cost in Australian dollars charged by Facebook for each user who clicks through the advertisement to the study landing website. CPC is related inversely to advertisement success, since a lower CPC indicates that more of the Facebook users that have been exposed to the advertisement, clicked on it.
2. Number and percentage of males completing an online expression of interest (EOI) in response to specific advertising strategies.
3. Number and percentage of males formally enrolled into the study (completing all baseline assessments and being in a cluster of 3–8 friends who have done the same) in response to specific advertising strategies.

### Procedure

Prior to conducting this study we disseminated initial recruitment campaigns to Facebook users who met study eligibility criteria. These standard campaigns were designed using Facebook’s Ads Manager, ran for four weeks, and consisted of images of either the study logo or a man wearing activewear tying his shoelaces, accompanied by a brief description study (see Additional file 1). All images used in this and subsequent campaigns were purchased from a stock image website (istock.com). This campaign generated 127 EOIs at a mean CPC of $AUD0.39. From this campaign, 55 participants formally enrolled into the study (AUD$11.36 per enrolled participant), but only 12.0% of these were men. Upon realising the gender imbalance, we took steps to redress this imbalance, which are reported in this research study. Our efforts to increase male recruitment fall into four distinct phases.

### Phase one: baseline campaign

In phase one we attempted to redress our gender imbalance by splitting our advertising budget to target two audience types (1) men and women together and (2) men only. A two-week campaign that used these audience parameters was conducted in early 2017. The male audience campaign was edited slightly to include the use of male pronouns and images of men, and was run alongside standard advertisements targeting men and women together. Both campaigns had equal budgets.

### Phase two: narrative literature review

We also conducted a narrative literature review of academic and grey literature with the aim to identify principles to guide the design of optimal Facebook-delivered health promotion content targeting men. Searches of the academic databases ProQuest, CINAHL, PubMed, ScienceDirect, and Google Scholar revealed a number of studies related to health promotion for men, but limited information related to effective Facebook advertisement design. Therefore, this search was extended to online grey literature identified using Google’s search engine.

### Phase three: audience survey

In phase three we aimed to identify advertisement components that were likely to be most appealing to the target audience of insufficiently active men. Drawing upon the marketing principles identified in phase two, ten pilot advertisements were created with each consisting of one image accompanied by up to 500 characters of text. A survey instrument containing 25 questions was developed and disseminated to participants representing our target audience, i.e., inactive Australian males aged between 18 and 65 years old. Within the survey, 20 items asked participants to rate the ten advertisement images and captions on a 10-point rating scale, with one being the most negative rating and ten being the most positive rating. Participants were also given the option to provide open-ended feedback about each advertisement. The survey also captured participants’ sociodemographic characteristics (e.g. age, sex), current physical activity level (days per week of > 30 min of MVPA, [19]), and physical activity stage of change according to the Trans theoretical Model, i.e. pre-contemplation, contemplation, preparation, action or maintenance [20]. The web link to the online survey was disseminated over one week in March 2017 using free techniques on Facebook, including sharing the link on the authors’ personal Facebook accounts with a brief message explaining the aim of the survey, eligibility criteria, and the expected completion time. Similar posts were made in Facebook community group pages and readers were encouraged to share the link.

The survey was completed by 74 respondents, however, during analysis it was noted that a high proportion of participants were highly active and self-reported to be in the maintenance phase of physical activity behaviour change. Since Active Team targets inactive adults, it was decided to focus analyses on participants in the pre-contemplation, contemplation, preparation, or action stages of change. Thus, 34 participants are included in the current analysis. Participants’ mean age was 30 (± 13.4) years old, and the majority (50%) had completed education to a high school level or less. On average, participants had completed 30 min of physical activity on a mean of three days in the past week (± 2.04).

### Phase four: evidence-based recruitment campaign

In phase four we incorporated results from phases one to three to develop and evaluate an evidence-based recruitment campaign delivered via Facebook. A Facebook campaign consisting of five advertisements using various combinations of the more highly ranked images and captions (total = five combinations; see Fig. 2) was developed. Campaign effectiveness was judged based on CPC, number of registrations (% men), and number of male enrolments into the study. The advertising campaign ran for one week in April 2017 and had a total budget of $AUD1000 that was distributed equally between the five advertisements ($AUD200 per advertisement). The parameters of the campaign were set to show the advertisements to Australian males aged 18 to 65 years.

## Results

### Phase one: baseline campaign

The CPC for ads targeting men and women together remained stable from pre-study campaigns at $AUD0.39, whereas the CPC for male-targeted ads was $AUD0.67. As a result of this campaign, 464 people (10.6% males) registered their interest to participate and 106 of these went on to enrol formally in the study (16.8% males). It was not possible to determine which EOIs and study enrolments were derived from which campaign. Altering Facebook parameters to disseminate advertisements to men only and minimally editing advertisement content increased the portion of men in our sample from 12.0 to 17.9%, indicating that this strategy was unlikely to be sufficient to redress the sex imbalance in the RCT.

### Phase two: narrative literature review

Literature reviews yielded information regarding health promotion for men and the design of Facebook advertising content (see Additional file 2 for more detail). According to this literature, health advertisement captions that focus on themes related to strength [17] and are positively framed i.e., that convey the health benefits of programs [21] are likely to be most effective. In terms of image selection, the literature suggests that portrayals of women may help to attract the attention of males [22], whilst portrayals of idealised (i.e., muscular, slender) male bodies may lead to feelings of poor body satisfaction in males and should be avoided [23]. Additional principles for the design of Facebook advertisements included that images should be visually engaging, attention grabbing, and thematically consistent with the product being advertised [24], captions should include a ‘low friction’ call-to-action (e.g., call for an expression of interest rather than immediate enrolment) [25], and, in cases where the promoting organisation is reputable, their branding should be clearly visible to convey scientific legitimacy [26].

### Phase three: audience survey

**Fig. 1:**
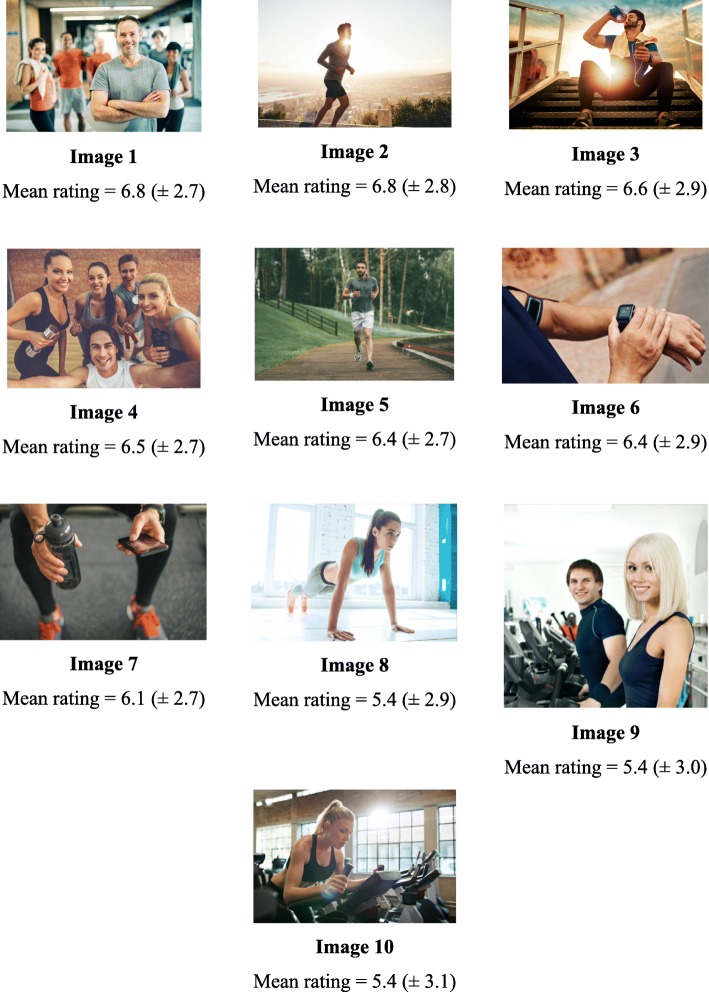
Mean rankings of advertisement images included in survey. All images were purchased from istock.com and the first author retains permission to reproduce them

Participants’ mean ratings of pilot images ranged from 5.4 (± 3.0) to 6.8 (± 2.7) out of a maximum of ten. The three highest-rated images (image 1–3 in Fig. 1) similarly portrayed a man in the foreground of the image, with the top ranked image displaying a man standing with arms crossed in front of a group of people dressed in active wear. Open-ended feedback suggested that this image was appealing to participants due to its friendly tone, with one participant describing it as “encouraging and relatable”. On the other hand, the main three lowest-rating images (images 8–10 in Fig. 1) all featured a female. Feedback indicated that these images were rated poorly because they sent a confusing message, with one participant asking, “why is there a female?” and another commenting that “this is very misleading as one would think this ad is for females only.”

Participants’ mean ratings of pilot text captions ranged from 5.2 (± 2.9) to 6.7 (± 2.7) out of a maximum score of ten (see Table 1). Open-ended responses suggested that concise captions (ranging between 18 and 35 words) were most appealing to participants, with one participant describing these as “simple, straight to the point” and “very smart”. On the other hand, the three lowest-rated captions were longer (between 41 and 85 words in length), and one participant described the lowest-rated caption as containing “too much information, (*I am*) not interested.”

**Table 1:** Mean ratings of captions included in pilot survey

Caption	Mean & standard deviation of ratings
Health researchers at UniSA have developed a smartphone app introducing a new way of tracking exercise to make life easier. It’s simple and quick to get started. Gather some friends and try it for FREE.	6.7 ± 2.7
Join the Active Team at UniSA. Health researchers at UniSA are developing a smartphone app to make increasing your physical activity simple. It’s a team effort, get your mates onto it.	6.3 ± 2.9
A call to MALES to test UniSA’s new fitness app. Be the first step in changing physical activity.	6.2 ± 2.8
MEN: Reduce your risk of stroke, cancer, and heart disease. Try UniSA’s newly developed app to see how physical activity can greatly reduce these health risks. With the expertise of health researchers at UniSA, you can make the change.	6.1 ± 2.8
Calling all blokes! Do you feel a bit out of shape? Do you like to set a good challenge for your mates? Are you between the ages of 18 – 65 yrs.? Health researchers at UniSA have developed a brand-new smartphone app called Active Team. It aims to promote physical activity by encouraging you to gather up some mates and try and tackle 10,000 steps a day for 100 days. Sound like a challenge? Take that first ‘step’ and click below to find out more.	6.0 ± 2.9
Guys! Are you looking to get in shape? Researchers at UniSA have developed a brand new smartphone app to help you do just that. Active Team promotes physical activity by encouraging you to take 10,000 steps a day. It’s quick, fun, and social – encouraging you to recruit friends and form teams so you can take on the challenge together. Are you up to the challenge? Click below to find out more.	6.0 ± 3.0
Male? Spend too much time on Facebook and not enough time exercising? Between the ages of 18 – 65 yrs.? UniSA may have developed the app for you. Health researchers at UniSA have recently developed Active Team, the smartphone app designed to help you get you active. The app sets the challenge of taking 10,000 steps a day. It encourages getting some friends together and taking on the challenge together. We are currently looking for males to help test the app. Take that first “step” and click below to find out more.	5.9 ± 2.9
We are looking for MEN to test a new app. Active Team is an app developed by UniSA’s health researchers to make exercising more social, simple, and fun. All female positions have been filled, we need MEN to join the team.	5.9 ± 2.8
Health researchers at UniSA have developed the exciting new smartphone app – Active Team. It encourages staying active through rounding up some mates and tackling the challenge of 10,000 steps a day. We are currently looking for males to help test the app. The first challenge… clicking below to find out more!	5.4 ± 2.7
A call to all guys – Are you currently physically inactive? Between the ages of 18 – 65 yrs.? Looking for that next challenge for you and your friends to try? How does 10,000 steps a day sound? Health researchers at UniSA have developed a brand-new smartphone app called – Active Team. The app promotes physical activity by setting the bar at 10,000 steps a day. It encourages forming teams to help take on the challenge together. Are you up to the challenge? Click below to find out more.	5.2 ± 2.9

*Note:* Captions are ordered from highest mean rating to lowest mean rating (top to bottom)

### Phase four: evidence-based recruitment campaign

**Fig. 2:**
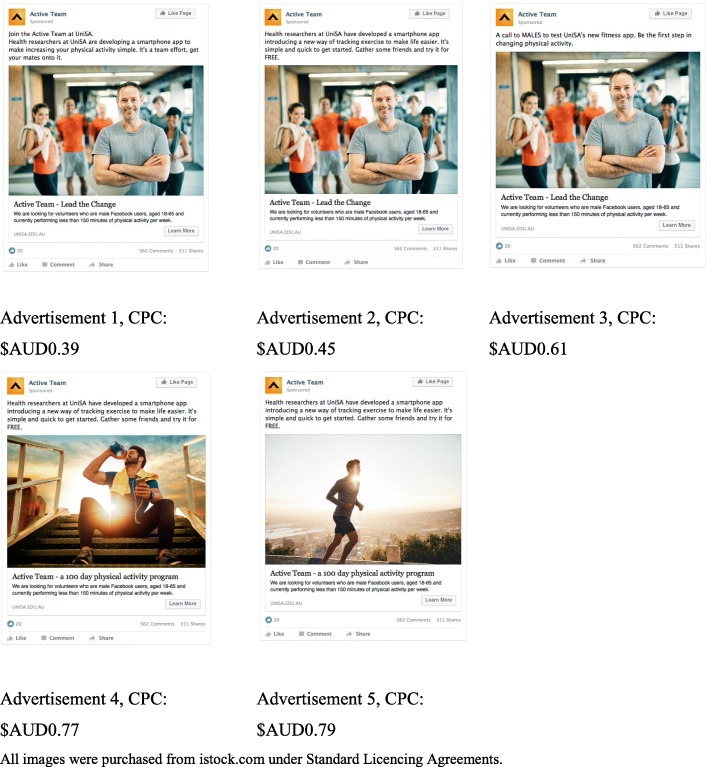
Pilot advertisements and cost-per-click (CPC) in pilot campaign. All images were purchased from istock.com and the first author retains permission to reproduce them

The evidence-based advertising campaign generated a total of 1665 clicks through to the landing page, with a mean CPC of $AUD0.60 (range = $0.39 to $0.79). The most successful advertisement contained the top ranked image with the second ranked caption from phase three, and the three most successful advertisements (lowest CPC) all contained the same image (see Fig. 2, image 1), suggesting that image may be more important than caption in eliciting audience engagement. The mean CPC for all ads in this campaign ($AUD0.60) was lower than that achieved in the baseline male-only campaign undertaken in phase one ($AUD0.67) and in fact, the highest performing advertisement achieved exactly the same CPC ($AUD0.39) as the mixed-gender campaign ($AUD0.39). In total, the evidence-based advertising campaign generated 50 EOIs, 80% of which were from men.

Despite these successes in terms of lowered CPC and expressions of interest, none of the men recruited through phase four continued on to successfully enrol in the study. This occurred despite the fact that research personnel followed up with each participant by telephone and email on three separate occasions, typically about one week apart. The most common reason for failing to enrol in the study after submitting an EOI was reluctance or inability to form an eligible team of between three to eight people. During our communications with these prospective participants, many expressed that they either felt uncomfortable asking others to join or could not think of anyone who would be interested in joining. Many asked to be placed in a team with others they didn’t know, which was not possible due to the requirement for teams to consist of existing friends. This experience differed from phase one when 20% of male EOIs successfully enrolled. In addition, some of the men recruited through phase four did form or join an eligible team but failed to enrol because they did not complete the baseline assessments. Anecdotally, prospective male participants recruited in phase one also required a greater number of follow-up contacts (e.g., phone calls, emails) to prompt them to complete these assessments and were more likely than female participants to fail to complete these requirements and withdraw prior to enrolment.

Upon consideration of results from the male-targeted campaign (phase four), we employed an alternative strategy to recruit men. During the process of following up EOIs from women, research personnel encouraged female participants to invite men from within their social networks to join Active Team. This practice led to an increase in the portion of males in the sample to 29.2%, which is greater than achieved in the original recruitment process that enrolled only 17.9% males. Overall, 107 out of 433 participants enrolled into the RCT to date are men (24.7%).

## Discussion

This paper set out to share details of our efforts and experiences recruiting men to an online physical activity program using Facebook advertising. Our experience suggests that men are markedly less likely to respond to unisex ads compared with women. The response rate from men was improved substantially through use of targeted Facebook advertising incorporating evidence-based features, such as using images of men that appealed to leadership themes, and using concise text. Piloting of the potential advertisements was useful for identifying the images and text that were most appealing to our target audience. Despite our success in improving men’s engagement with study recruitment advertisement, the evidence-based campaign alone did not culminate in improved enrolments although we were able to boost male enrolments by asking female participants to invite males.

This study uncovered a number of principles relating to the design of effective health promotion advertisements targeting men via Facebook. Firstly, image selection may be more influential than text caption when it comes to stimulating audience engagement and click-throughs. One image that depicted a theme of male leadership and had a positive and friendly tone stood out as being particularly effective in attracting audience attention. This is consistent with previous research that found positively-framed appeals, particularly those that promote positive masculine norms (e.g. strength), to be most effective in engaging males with health programs [27]. Secondly, despite research that suggests sex appeal is effective in attracting males’ attention [22], our survey respondents reported that images featuring females were the least appealing and sent a confusing message. Consistent with the schema congruity hypothesis, this may reflect the human tendency to seek out information in our environment that is thematically consistent in order to minimise cognitive processing [28]. As previously suggested [29, 30], future campaigns should strive to present a clear, cohesive message that is consistent through both the text caption and the image. Finally, although Facebook does not limit the length of advertisement captions, target audience members showed a clear preference for briefer captions (< 35 words). This finding may reflect the message-dense nature of OSN content, often containing a myriad of information including advertisements, user-generated content, and promoted posts that make it difficult to grab the attention of users. It is therefore of utmost importance that health messages on OSNs are immediately understandable and brief enough (i.e., < 35 words) to be read and interpreted quickly.

Difficulties recruiting males to health behaviour research are not uncommon, with physical activity interventions that target both males and females reporting much higher rates (> 80%) of female participation [7]. In the current study we observed that many of the males who did click through to our landing page and complete some or all of the baseline assessments did not go on to complete their formal enrolment in the study. This phenomenon may be a result of the more intensive nature of the physical activity intervention that also had a relatively long timeframe of commitment (9 months). Participants are required to complete assessments over a nine-month period, and being a three-arm RCT, participants’ chance of random allocation to the treatment arm is just 33%. Previous studies have similarly suggested that Facebook advertising may be better suited to recruiting participants to less intensive or once-off data collection rather than ongoing participation in intervention studies [31, 32].

There was also some anecdotal evidence to suggest that the social networking nature of Active Team was more appealing to women than it was to men. The requirement to form a team of three to eight existing Facebook friends appeared to be a significant barrier to the enrolment of males, but not females, into the study. A similar experience was reported in the ManUp RCT, which sought to improve physical activity and dietary practices in middle-aged men using a Web-based approach that included an intentionally designed social network [33]. Of the 214 participants that were randomised to the OSN component, only 21 participants elected to find a ‘mate’ and no participants had more than one mate in this network. It is possible that males are more inclined to participate in individual-based physical activity programs rather than social ones, or perhaps they would prefer to interact with strangers rather than people they know. Lifestyle, use of time patterns, and physical activity preferences are also likely determinants of male participation in health behaviour research. Although the bulk of physical activity interventions are based on a target of 10,000 steps per day, research suggests that males may prefer a more diverse range of activities including walking, swimming, cycling, and team sports [34]. In addition, males are more likely to partake in full time employment than women [35], and this may limit opportunities to accumulate step counts throughout the day via incidental activity. For these reasons, interventions that promote time-based goals, such as 30 min of activity per day rather than 10,000 steps per day, may be more appealing to men [34].

There are a number of limitations that are worth considering when interpreting these findings. Firstly, our Facebook advertisements were displayed to different audiences during each of the different phases (i.e., results reflect an open cohort design). Although this characteristic of the study design was necessary as a means to test the advertisements in real-life conditions, it is important to acknowledge that conclusions are correlational rather than causal. Secondly, online survey respondents were recruited via the authors’ personal Facebook pages and local Facebook interest groups, which may have created some biases in our sample towards users who are highly engaged with their community and health. Finally, the target population in this study was broad and included adults aged 18–65, which may have shrouded any effects of age on responses to health information. Further research is needed to explore how age, and other individual differences including socio-economic status and personality affect responses to health information even within the male sub-group.

Whilst not a direct limitation of the current study, there are challenges associated with the use of Facebook advertising that should be briefly mentioned here. There is little information available about the algorithm Facebook uses to determine the amount of exposure that an ad receives. The algorithm controls the numbers of ads that a user sees, and the content of the ads based on a combination of factors including the parameters set by the advertiser, previous engagement with the ad (likes, comments, and shares).

## Conclusions

Under-recruitment of males is a general limitation of health behaviour research. Since the typical research participant in health behaviour research is a university-education, middle-aged woman, conclusions about men may be underpowered or difficult to generalise. In light of evidence that males are less likely to seek help for health-related issues [36] and less likely to participate in health promoting programs [3], expanding our understanding of how to effectively promote health programs to men is an essential avenue for future research. The current study provides some valuable insights into how recruitment rates for male participants to physical activity programs can be improved. Despite careful development and implementation, an evidence-based Facebook advertisement campaign was not enough to address the gender imbalance within the RCT sample, however, the approach of encouraging female participants to invite male participants was much more successful, and resulted in a 66% relative increase in male enrolment.

## Additional files

Additional file 1: Sample advertisements from the baseline advertising campaign: advertisement targeting men and women together (left) and male-targeted advertisement (right). (DOCX 395 kb)

Additional file 2: Marketing principles relating to the design of Facebook advertisements to attract men to health programs [37–39]. (DOCX 15 kb)

## Data Availability

The datasets used and analysed during the current study are available from the corresponding author on reasonable request.

## Abbreviations

App: mobile phone application
CINAHL: Cumulative Index to Nursing and Allied Health Literature
CPC: Cost per click
EOI: Expression of interest
OSN: Online social network
RCT: Randomised controlled trial

## References

[1] World Health Organisation. Global Recommendations on Physical Activity for Health. Geneva; 2010. https://www.who.int/dietphysicalactivity/factsheet_recommendations/en/. 26180873

[2] Hallal PC, Andersen LB, Bull FC, Guthold R, Haskell W, Ekelund U (2012). Global physical activity levels: surveillance progress, pitfalls, and prospects. Lancet.

[3] Mahalik JR, Burns SM, Syzdek M (2007). Masculinity and perceived normative health behaviors as predictors of men's health behaviors. Soc Sci Med.

[4] Noble N, Paul C, Turon H, Oldmeadow C (2015). Which modifiable health risk behaviours are related? A systematic review of the clustering of smoking, nutrition, alcohol and physical activity (‘SNAP’) health risk factors. Prev Med.

[5] Yousaf O, Grunfeld EA, Hunter MS (2015). A systematic review of the factors associated with delays in medical and psychological help-seeking among men. Health Psychol Rev.

[6] Rongen A, Robroek SJ, van Lenthe FJ, Burdorf A (2013). Workplace health promotion: a meta-analysis of effectiveness. Am J Prev Med.

[7] Maher Carol A, Lewis Lucy K, Ferrar Katia, Marshall Simon, De Bourdeaudhuij Ilse, Vandelanotte Corneel (2014). Are Health Behavior Change Interventions That Use Online Social Networks Effective? A Systematic Review. Journal of Medical Internet Research.

[8] Robertson LM, Douglas F, Ludbrook A, Reid G, van Teijlingen E (2008). What works with men? A systematic review of health promoting interventions targeting men. BMC Health Serv Res.

[9] Dooley JA, Jones SC, Iverson D (2014). Using web 2.0 for health promotion and social marketing efforts: lessons learned from web 2.0 experts. Health Mark Q.

[10] Vandelanotte C, Müller AM, Short CE, Hingle M, Nathan N, Williams SL (2016). Past, present, and future of eHealth and mHealth research to improve physical activity and dietary behaviors. J Nutr Educ Behav.

[11] Alley S, Wellens P, Schoeppe S, de Vries H, Rebar AL, Short CE (2017). Impact of increasing social media use on sitting time and body mass index. Health Promot J Austr..

[12] Facebook. Stats 2017 [Available from: https://newsroom.fb.com/company-info/.

[13] Statista. Distribution of Facebook users worldwide as of January 2017, by age and gender 2017 [Available from: https://www.statista.com/statistics/376128/facebook-global-user-age-distribution/.

[14] Thornton L, Batterham PJ, Fassnacht DB, Kay-Lambkin F, Calear AL, Hunt S (2016). Recruiting for health, medical or psychosocial research using Facebook: systematic review. Internet Interv.

[15] Whitaker Christopher, Stevelink Sharon, Fear Nicola (2017). The Use of Facebook in Recruiting Participants for Health Research Purposes: A Systematic Review. Journal of Medical Internet Research.

[16] Amon KL, Campbell AJ, Hawke C, Steinbeck K (2014). Facebook as a recruitment tool for adolescent health research: A Systematic Review. Acad Pediatr.

[17] Choi I, Milne DN, Glozier N, Peters D, Harvey SB, Calvo RA (2017). Using different Facebook advertisements to recruit men for an online mental health study: engagement and selection bias. Internet Interv.

[18] Edney S, Plotnikoff R, Vandelanotte C, Olds T, De Bourdeaudhuij I, Ryan J, Maher C (2017). “Active team” a social and gamified app-based physical activity intervention: randomised controlled trial study protocol. BMC Public Health.

[19] Milton K, Bull F, Bauman A (2011). Reliability and validity testing of a single-item physical activity measure. Br J Sports Med.

[20] Cook CL, Perri M (2004). Single-item vs multiple-item measures of stage of change in compliance with prescribed medications. Psychol Rep.

[21] Li K-K, Cheng S-T, Fung HH (2014). Effects of message framing on self-report and accelerometer-assessed physical activity across age and gender groups. J Sport Exerc Psychol.

[22] Black IR, Morton P (2017). Appealing to men and women using sexual appeals in advertising: in the battle of the sexes, is a truce possible?. J Mark Commun.

[23] Galioto R, Crowther JH (2013). The effects of exposure to slender and muscular images on male body dissatisfaction. Body Image.

[24] Bernazanni S. 2016. [cited 2017 7 March]. Available from: https://blog.hubspot.com/blog/tabid/6307/bid/33319/10-examples-of-facebook-ads-that-actually-work-and-why.aspx#sm.000086kl84nhscpfsq62d22487j1g.

[25] Porretta F. 2012. [cited 2017 6 March]. Available from: https://blog.kissmetrics.com/deep-dive-facebook-advertising/.

[26] Morgan Amy Joanna, Jorm Anthony Francis, Mackinnon Andrew James (2013). Internet-Based Recruitment to a Depression Prevention Intervention: Lessons From the Mood Memos Study. Journal of Medical Internet Research.

[27] Bottorff JL, Seaton CL, Johnson ST, Caperchione CM, Oliffe JL, More K (2015). An updated review of interventions that include promotion of physical activity for adult men. Sports Med.

[28] Meyers-Levy J, Tybout AM (1989). Schema congruity as a basis for product evaluation. J Consum Res.

[29] Chapman C. 2011. [cited 2017 March 7]. Available from: https://blog.kissmetrics.com/beginners-guide-to-landing-pages.

[30] Partridge Stephanie R, Balestracci Kate, Wong Annette TY, Hebden Lana, McGeechan Kevin, Denney-Wilson Elizabeth, Harris Mark F, Phongsavan Philayrath, Bauman Adrian, Allman-Farinelli Margaret (2015). Effective Strategies to Recruit Young Adults Into the TXT2BFiT mHealth Randomized Controlled Trial for Weight Gain Prevention. JMIR Research Protocols.

[31] Batterham PJ (2014). Recruitment of mental health survey participants using internet advertising: content, characteristics and cost effectiveness. Int J Methods Psychiatr Res.

[32] Ellis LA, McCabe KL, Rahilly KA, Nicholas MA, Davenport TA, Burns JM (2014). Encouraging young men's participation in mental health research and treatment: perspectives in our technological age. Clin Investig.

[33] Duncan Mitch, Vandelanotte Corneel, Kolt Gregory S, Rosenkranz Richard R, Caperchione Cristina M, George Emma S, Ding Hang, Hooker Cindy, Karunanithi Mohan, Maeder Anthony J, Noakes Manny, Tague Rhys, Taylor Pennie, Viljoen Pierre, Mummery W Kerry (2014). Effectiveness of a Web- and Mobile Phone-Based Intervention to Promote Physical Activity and Healthy Eating in Middle-Aged Males: Randomized Controlled Trial of the ManUp Study. Journal of Medical Internet Research.

[34] Burton NW, Walsh A, Brown WJ (2008). It just doesn't speak to me: mid-aged men's reactions to'10,000 steps a Day'. Health Promot J Aust.

[35] OECD. Usual working hours per week by gender. Directorate of Employment, Labour and Social Affairs; 2013.

[36] Galdas PM, Cheater F, Marshall P (2005). Men and health help-seeking behaviour: literature review. J Adv Nurs.

[37] Jancey J, Howat P, Lee A, Clarke A, Shilton T, Fisher J, Iredell H (2006). Effective recruitment and retention of older adults in physical activity research: PALS study. Am J Health Behav.

[38] Lane Taylor S, Armin Julie, Gordon Judith S (2015). Online Recruitment Methods for Web-Based and Mobile Health Studies: A Review of the Literature. Journal of Medical Internet Research.

[39] Torr, D 2017, ‘How to advertise on Facebook: a beginner’s guide’, blog post, Hootsuite, viewed 6 March 2017, <https://blog.hootsuite.com/how-to-advertise-on-facebook/>.

