# Supplementary material for: It’s not raining men: a mixed-methods study investigating methods of improving male recruitment to health behaviour research
Source: BMC Public Health. 2019 Jun 24;19:814. doi: 10.1186/s12889-019-7087-4 (PMC6591998; doi:10.1186/s12889-019-7087-4)
Supplement: Supplementary file 1 — Sample advertisements from the baseline advertising campaign: advertisement targeting men and women together (left) and male-targeted advertisement (right). (DOCX 395 kb) [file 12889_2019_7087_MOESM1_ESM.docx]

| 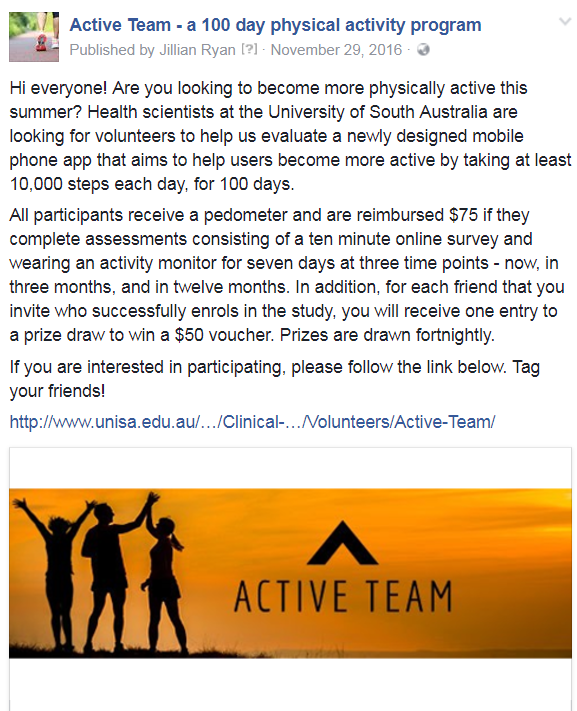 | 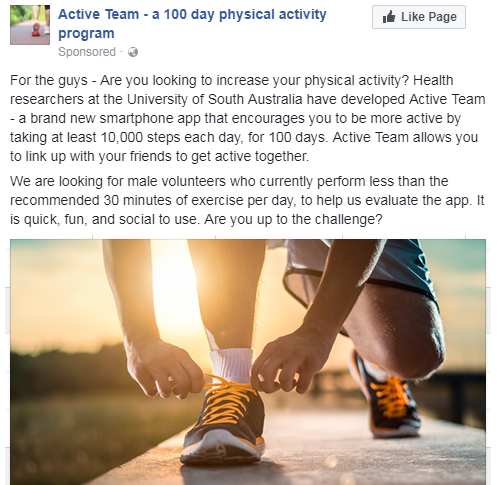 |
| --- | --- |

*Figure 1.*

Sample advertisements from the baseline advertising campaign: advertisement targeting men and women together (left) and male-targeted advertisement (right).
