# Supplementary material for: It’s not raining men: a mixed-methods study investigating methods of improving male recruitment to health behaviour research
Source: BMC Public Health. 2019 Jun 24;19:814. doi: 10.1186/s12889-019-7087-4 (PMC6591998; doi:10.1186/s12889-019-7087-4)
Supplement: Supplementary file 2 — Marketing principles relating to the design of Facebook advertisements to attract men to health programs [37–39]. (DOCX 15 kb) [file 12889_2019_7087_MOESM2_ESM.docx]

| Supplementary Table 1. Marketing principles relating to the design of Facebook advertisements to attract men to health programs | |
| --- | --- |
| Principles related to Facebook marketing in general | - Advertisement should strive to elicit Attention, Interest, Desire, and Action (AIDA) (25). - All components of the advertisement (e.g. images, captions, and linked webpages), should follow a consistent theme that aligns with a broader campaign or brand (29). - Promoting organisation’s logo should be displayed in a prominent position (26). |
| Principles related to targeting men | - Advertisements featuring themes of masculinity and strength (e.g. asking the question, “How tough is your mind?”) are likely to appeal more to men than other themes such as happiness and resilience (17). - Advertisements portraying the opposite sex may be appealing and help to attract the attention of men (22). - Promoting the health benefits of PA more likely to appeal to older men (21). |
| Principles related to selection of images | - Image should be visually engaging, colourful and memorable (24), and thematically consistent, for example, depict individuals performing physical activity (30), or show relevant sporting equipment (37). - Image size should 1200 wide and 680 high in order to be of high resolution quality (Kolowich 2017), and to comply with Facebook’s image restrictions (Bernazanni 2016). - Image should be original/unique, rather than obviously resembling stock imagery (25). |
|  |  |
|  |  |
| Principles related to the framing of text captions | - Text should be informative (38) and utilise appropriate language that is appropriate for the target audience’s demographic characteristics (e.g. age, education level), and interests (39). - Text should use wording that promotes problem solving, e.g., ask a question or make a value proposition or call-to-action (25, 37, 38). - Advertisements should use a low friction conversation method to promote expressing interest, rather than directly enrolling in a study (25). |
